# Comparison of intradialytic versus home-based exercise programs on physical functioning, physical activity level, adherence, and health-related quality of life: pilot study

**DOI:** 10.1038/s41598-020-64372-y

**Published:** 2020-05-19

**Authors:** Lucía Ortega-Pérez de Villar, Francisco José Martínez-Olmos, Francisco de Borja Pérez-Domínguez, Vicent Benavent-Caballer, Francisco Javier Montañez-Aguilera, Tom Mercer, Eva Segura-Ortí

**Affiliations:** 1 Department of Physical Therapy, Universidad Cardenal Herrera-CEU, CEU Universities, Valencia, Spain; 2 Department of Physical Therapy, Universidad Europea de Valencia, Valencia, Spain; 3 Centre for Health, Activity and Rehabilitation Research School of Health Sciences, Queen Margaret University, Musselburgh, UK

**Keywords:** Quality of life, Rehabilitation, End-stage renal disease

## Abstract

Intradialytic exercise (ID) programs are effective and safe for hemodialysis (HD) patients to avoid functional deterioration. However, exercise is not routinely undertaken in most HD units, and we do not know if home-based (HB) programs are as effective as ID programs. The purpose of this study was to compare the effects of 16 weeks of ID exercise versus a HB exercise program for HD patients. A total of 46 patients were randomly assigned to the ID group (*n* = 24) or HB group (*n* = 22). They completed a 16-week combined exercise program 3 times/week. We measured physical activity level, physical functioning, depression level, and health-related quality of life at baseline and after 16 weeks. A significant time effect was found in both groups for the physical activity level (*p* = 0.012). There was also a significant group–time interaction effect for the one-leg standing test (OLST) (*p* = 0.049) and a significant time effect for the Short Physical Performance Battery (*p* = 0.013), timed up-and-go test (*p* = 0.005), sit-to-stand-10 (*p* = 0.027), right and left hand handgrip (*p* = 0.044, *p* < 0.001), one-heel left leg raise (*p* = 0.019), and 6-minute walking (*p* = 0.006), depression (p = 0.017). HRQoL remained unchanged. There was no difference between the two interventions on the tested outcomes (besides OLST). Both interventions were associated with positive changes of the physical activity levels and physical function.

## Introduction

Chronic kidney disease (CKD) patients undergoing hemodialysis (HD) present a progressive functional deterioration, poor health-related quality of life (HRQoL), and low levels of physical activity compared to their healthy age-matched counterparts^1,2^. In addition, this population often presents symptoms of depression and anxiety^3–5^. Exercise programs for people with CKD have been implemented since the beginning of 1980s^6^. For optimal benefits, these programs should include a combination of aerobic exercise and strength resistance^7^. The few studies carried out in HD units in Spain have tested different exercise types including strength-resistance^8^, resistance^9^, low intensity endurance training^10,11^, neuromuscular electrostimulation^12,13^, and virtual reality^14^.

Different exercise modalities have been studied in the literature, but intradialytic (ID) exercise is considered the best approach^15^ because adherence to this modality is higher than with other formats^16^. Nevertheless, to the best of our knowledge, HD units in Spain do not currently include exercise as part of routine HD treatments, perhaps because of the financial cost associated with ID exercise programs. Thus, home-based (HB) programs may be an option that could compensate for these cost-related constraints^17^. To our knowledge, only a few studies have applied HB exercise programs. We found a total of twelve HB studies in HD patients^17–28^: 9 of them were randomized controlled trials^16–18,23–28^, and only 3^16–18^ implemented a combined HB exercise program comprising both aerobic and strength exercises. Several studies measured physical functioning through physical performance tests but none analyzed the effect of the interventions on physical activity levels.

It is well known that the physical activity levels of patients in maintenance HD are lower than their healthy age-matched counterparts^29–31^. Sedentary behavior is associated with physical deterioration and usually results in limitations in the performance of the activities of daily living which could lead to disability^32–34^. This can increase both the number of comorbid conditions presented by patients with HD as well as their mortality risk (especially with cardiovascular problems)^35^. Thus, it is important to implement interventions to increase the physical activity level of patients with HD.

Therefore, the primary aim of this study was to compare the effects of ID versus HB exercise programs on the physical activity level of patients on maintenance HD. The secondary aims were (i) to compare the effects of ID versus HB exercise on physical function, (ii) HRQoL, (iii) the symptoms of depression, and (iv) on adherence. We hypothesized that the ID program would result in more benefits than the HB exercise program.

## Results

**Figure 1:**
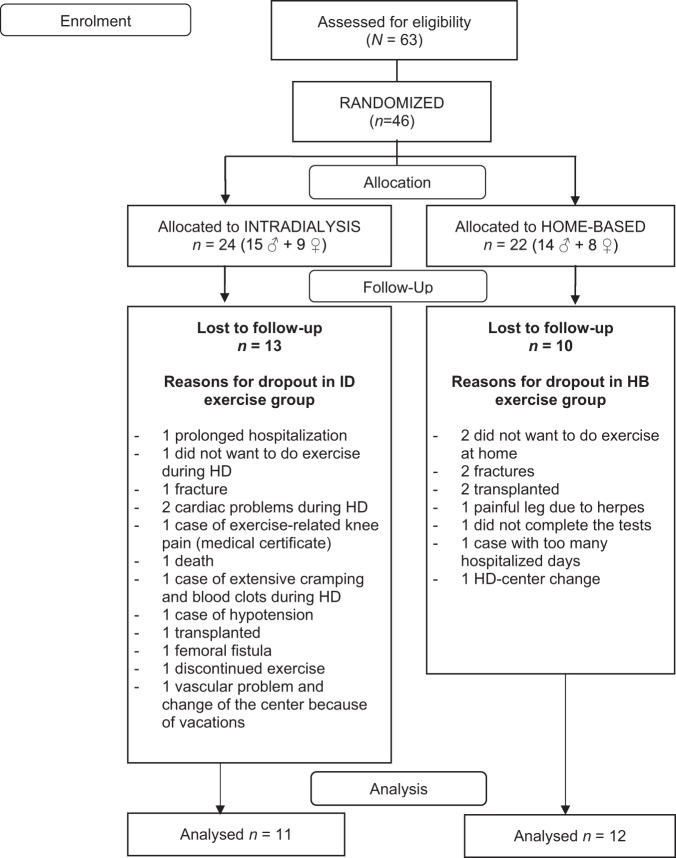
Flow chart depicting the progression of the participants completing the intradialytic versus home-based exercise programs through the trial. Patient adherence and dropouts are shown.

Forty-six patients undergoing HD were recruited and randomly allocated into the ID or HB exercise groups. There were 13 dropouts in the ID group and 10 in the HB group (see Fig. 1). Table 1 shows the baseline clinical characteristics and demographics of the study cohort. No significant differences were found between groups at baseline either in demographic or functional tests, indicating the successful randomization of the participants.

**Table 1:** Baseline clinical characteristics and demographic data (*n* = 46).

Variable	ID exercise	ID exercise	HB Exercise	HB exercise
	Intention to treat (*n* = 24)	Per-protocol (n = 11)	Intention to treat (*n* = 22)	Per-protocol (n = 12)
Age (years)				
Mean (*SD*)	62.2 (15.0)	65.3 (15.2)	59.3 (16.1)	61.9 (12.1)
Median (min–max)	66.5 (45.8–72.3)	69 (37–81)	57 (46–73)	65 (37–78)
Time on HD (months)				
Median (P25–P75)	46 (24–61)	40.6 (32.7)	45 (24–122)	89.8 (32.7)
Sex *n* (%)				
Male	15 (62.5)	15 (32.6)	14 (63.6)	14 (30.4)
Female	9 (37.5)	9 (19.6)	8 (36.4)	8 (17.4)
Weight, (kg)				
Mean (*SD*)	73.6 (13.9)	73.6 (13.9)	70.5 (17.1)	70.5(17.1)
Median (min–max)	71.5 (54.5–103)	71.5 (54.5–103)	67 (49–117)	67 (49–117)
Height, (cm)				
Mean (*SD*)	1.66 (0.1)	1.66 (0.1)	1.67 (0.1)	1.67 (0.1)
Median (min–max)	1.67 (1.5–1.8)	1.67 (1.5–1.8)	1.68 (1.5–1.8)	1.68 (1.5–1.8)
Body mass index (kg/m^2^)				
Mean (*SD*)	26.6 (3.7)	26.6 (3.7)	25.1 (5.3)	26.6 (3.7)
Median (min–max)	26.1 (21.3–32.9)	26.1 (21.3–32.9)	23.8 (17.6–37.8)	23.8 (17.6–37.8)
Albumin (mg/dL)				
Mean (*SD*)	3.6 (0.4)	3.6 (0.4)	3.7 (0.3)	3.7 (0.3)
Median (min–max)	3.6 (2.5–4.2)	3.6 (2.5–4.2)	3.7 (3.3–4.2)	3.7 (3.3–4.2)
Creatinine (mg/dL)				
Mean (*SD*)	6.7 (3.4)	6.7 (3 .4)	7.9 (3.9)	7.9 (3.9)
Median (min–max)	7.2 (3.0–12.7)	7.2 (3.0–12.7)	8.3 (3.1–17.4)	8.3 (3.1–17.4)
Hemoglobin (g/dL)				
Mean (*SD*)	10.8 (0.8)	10.8 (0.8)	10.5 (1.7)	10.5 (1.7)
Median (min–max)	10.8 (9.4–12.5)	10.8 (9.4–12.5)	11.1 (6.6–12.8)	11.1 (6.6–12.8)
CKD Diagnosis				
Diabetes mellitus	3	3	5	5
Glomerulonephritis	4	4	8	8
Lupus	3	3	0	0
Polycystosis	1	1	0	0
Nephroangiosclerosis	2	2	1	1
Pyelonephritis	1	1	1	1
High blood pressure	3	3	0	0
Traumatic kidney injury	0	0	2	2
Others	7	7	5	5
Charlson comorbidity score				
Mean (*SD*)	6.6 (2.8)	6.6 (2.8)	6.0 (2.0)	6.0 (2.0)
Median (min–max)	7 (1–12)	7 (1–12)	6 (3–10)	6 (3–10)

CKD: chronic kidney disease; SD: standard deviation.

Baseline data comparison between intention to treat [ID (n = 24) and HB (n = 22)] or per-protocol groups [ID (n = 11) and HB (n = 12)] with the Mann-Whitney and Chi squared test showed no significant differences.

The adherence to the exercise programs was 80.8% for the ID exercise group (number of sessions completed P25 = 29-P75 = 42 and number of missed session P25 = 6-P75 = 19) and 53% for the HB group (number of sessions completed P25 = 11 P75 = 37 and number of missed session HB P25 = 3-P75 = 37).

### Primary outcomes

Table 2 shows the physical activity values measured via the HAP. There was a significant time effect but a non-significant interaction effect and both groups had increased their physical activity levels by the end of the intervention (ID group pre = 62.4 ± 16.6, post = 67.3 ± 15.6; HB group pre=51.1 ± 18.1, post=54.3 ± 19.3; *F* = 7.642, *p* = 0.012). Another secondary measure of physical activity showed similar results (PASE results in Table 2).

**Table 2:** Mixed model of repeated measures for patient physical activity levels.

Variable	Group	Mean ± standard deviation		Analysis of variance (group × time), *p*-value	Effect size	Analysis of variance (time), p-value	Effect size
		Baseline	After 16 weeks				
HAP AAS (points) Mean (*SD*)	ID	62.4 (16.6)	67.3 (15.6)	*F* = 0.356, 0.557	0.017	***F*** = **7.642, 0.012**	0.267
	HB	51.1 (18.1)	54.3 (19.3)				
HAP MAS (points) Mean (*SD*)	ID	76.4 (8.2)	80 (8.5)	*F* = 1.501, 0.234	0.067	*F* = 3.890, 0.062	0.156
	HB	70.8 (11.6)	71 (15)				
PASE (points) Mean (*SD*)	ID	112.1 (113.7)	138.6 (113.1)	*F* = 0.041, 0.842	0.002	***F*** = **15.642, 0.001**	0.427
	HB	59.4 (39.9)	83.4 (53.1)				

HAP: Human Activity Profile questionnaire; MAS: the number assigned to the activity with the highest oxygen consumption requirement on the HAP questionnaire that the patient still performs; ASS: the adjusted activity score is the difference between the MAS and the number of activities with lower scores (less demanding tasks) on the HAP questionnaire that the participant has stopped performing; PASE: Physical Activity Scale for Elderly.

Baseline data comparison between ID (n = 11) and HB (n = 12) with the Mann-Whitney test showed no significant differences.

Both interventions were equally effective at increasing physical activity levels among the participants after 16 weeks of ID or HB exercise, without differences between groups. At baseline the HB group contained 8 individuals with impaired activity (AAS < 53 points), 1 who was moderately active (AAS = 53–74 points), and 3 active patients (AAS > 74 points), whereas at the end of the HB, 5 individuals were moderately active. Regarding the ID group, at baseline 5 individuals had impaired activity, 3 were moderately active, and 3 were active; by the end of the program 4 individuals were moderately active and 5 were active. Both interventions lifted participants from the impaired activity category.

### Secondary outcomes

Table 3 shows the values from the physical functioning tests for each group at baseline and after 16 weeks performing a combined exercise program. The results of the two-way ANOVA showed a significant group–time interaction for the OLST (*p* = 0.049, ${\eta }_{p}^{2}$ = 0.189). The within-group analysis showed significant improvements in the ID group for the OLST (5.9 s, 95%CI [1.42–10.55]; *p* = 0.013). The time–group interaction was not significant for the STS-10, SPPB, TUG, HG, or 6MWT. However, we observed a significant time effect, indicating that performance improved in both intervention groups.

**Table 3:** Mixed model of repeated measures for the physical functioning tests.

Variable	Group	Mean ± standard deviation		Analysis of variance (group × time), *p*-value	Effect size	Analysis of variance (time), *p*-value	Effect size
		Baseline	After 16 weeks				
STS-10 (seconds) Mean (*SD*)	ID	25.4 (10.6)	22.3 (7.2)	*F* = 0.726, 0.404	0.035	***F*** = **5.678, 0.027**	0.221
	HB	25 (10.7)	23.6 (8)				
SPPB (points) Mean (*SD*)	ID	10.6 (1.43)	11 (1.6)	*F* = 0.643, 0.432	0.030	***F*** = **7.433, 0.013**	0.261
	HB	10.1 (2.3)	10.8 (2.1)				
OLST (seconds) Mean (*SD*)	ID	12 (14.1)	17.9 (18.4)	***F*** = **4.421, 0.049**	0.189	*F* = 2.829, 0.109	0.130
	HB	17.2 (19.4)	16.6 (19.8)				
TUG (seconds) Mean (*SD*)	ID	7.9 (1.7)	7.6 (1.6)	*F* = 0.405, 0.531	0.019	***F*** = **9.717, 0.005**	0.316
	HB	9.5 (2.5)	9.1 (2.2)				
STS-60 (repetitions) Mean (*SD*)	ID	19.7 (7.4)	23.6 (8.2)	*F* = 3.911, 0.062	0.164	*F* = 1.795, 0.195	0.082
	HB	21.6 (7.7)	20.8 (3.7)				
HG R hand (Kg) Mean (*SD*)	ID	27.8 (11.8)	30 (9.4)	*F* = 0.546, 0.468	0.025	***F*** = **4.577, 0.044**	0.179
	HB	29.3 (11.7)	30.3 (11.1)				
HG L hand (Kg) Mean (*SD*)	ID	26.6 (9.7)	28.7 (9.6)	*F* = 2.061, 0.166	0.089	***F*** = **18.212, 0.000**	0.464
	HB	25.8 (11.5)	26.9 (10.7)				
One-Heel Raise R Leg (repetitions) Mean (*SD*)	ID	23.3 (2.5)	23.6 (3.6)	*F* = 0.000, 0.995	0.000	*F* = 0.144, 0.709	0.008
	HB	18.5 (9.2)	18.9 (9.4)				
One-Heel Raise L leg (repetitions) Mean (*SD*)	ID	21.6 (4.8)	24.7 (1)	*F* = 0.011, 0.919	0.001	***F*** = **6.862, 0.019**	0.314
	HB	17.3 (10.1)	20.1 (9.5)				
6MWT (meters) Mean (*SD*)	ID	410.7 (107.9)	436.2 (100.5)	*F* = 0.108, 0.745	0.005	***F*** = **9.314, 0.006**	0.307
	HB	360.7 (126.7)	381.2 (96.2)				

STS: the sit-to-stand test; SPPB: Short Physical Performance Battery; OLST: One-leg standing test; TUG: timed up-and-go test; HG: handgrip strength; 6MWT: 6 minutes walking test.

Baseline data comparison between ID (n = 11) and HB (n = 12) with the Mann-Whitney test showed no significant differences.

The values obtained for the CES-D and HRQoL in each group at baseline and after 16 weeks are shown in Table 4. The group–time interaction was not significant for any of the questionnaires. Depression rates improved in both intervention groups and HRQoL components remained unchanged.

**Table 4:** Mixed-model of repeated measures for depression and health-related quality of life.

Variable	Group	Mean ± standard deviation		Analysis of variance (group × time), *p*-value	Effect size	Analysis of variance (time), *p*-value	Effect size
		Baseline	After 16 weeks				
CES-D (points) Mean (*SD*)	ID	15.5 (13.2)	9.2 (8.7)	*F* = 3.370, 0.081	0.138	***F*** = **6.772, 0.017**	0.244
	HB	15.6 (9.9)	14.5 (8.1)				
Symptoms and problems list (points) Mean (*SD*)	ID	83.9 (12.5)	87.7 (11.3)	*F* = 2.375, 0.138	0.102	*F* = 0.738, 0.400	0.034
	HB	81.9 (10.7)	80.9 (12.4)				
Burden of kidney disease (points) Mean (*SD*)	ID	51.7 (29.1)	51.7 (31)	*F* = 0.163, 0.691	0.008	*F* = 0.163, 0.691	0.008
	HB	40.1 (15.9)	43.2 (22.7)				
Effects of kidney disease on daily life (points) Mean (*SD*)	ID	73.6 (16.8)	65.3 (23.9)	*F* = 0.392, 0.538	0.018	*F* = 2.625, 0.120	0.111
	HB	78.6 (19.1)	75 (21.2)				
Physical component summary (points) Mean (*SD*)	ID	42.0 (10.4)	45.7 (11.2)	*F* = 1.172, 0.291	0.053	*F* = 2.865, 0.105	0.120
	HB	40.9 (8.6)	41.8 (12.8)				
Mental component summary (points) Mean (*SD*)	ID	49.9 (9.5)	50.3 (8.1)	*F* = 0.772, 0.390	0.035	*F* = 1.177, 0.290	0.053
	HB	47 (10.3)	49.9 (11.1)				

CES-D: Center for Epidemiologic Studies Depression scale.

## Discussion

The purpose of this study was to compare the effect on HD patients of performing an ID versus HB 16-week exercise program. To our knowledge, this is the first HB exercise program devised and tested in Spain. We wanted to discover if, in a Spanish population on HD, performing a HB program improved patient physical activity, physical functioning, and HRQoL and CES-D scores.

The study design tried to implement two different interventions balanced in exercise intensity and volume. Both programs aimed at improving lower limb muscle strength of the most commonly used muscle groups in activities of daily living, although the restricted position for the ID group limited how to perform strengthening exercises, and at HB group was not restricted at all regarding positions to do strength exercises. We tried also to mimic the aerobic component, though ID group cycled while the HB group walked. Sessions per week, exercise volume (sets and repetitions, so as weights lifted) and exercise time was quite similar in both groups.

Increased physical activity levels may result in a better physical condition^3^. Thus, we believe the increase we found is important and hope it will inspire nephrologists and renal nurses to implement interventions designed to encourage exercise among patients in HD units. A previous study did not find any significant differences between 12 weeks of resistance training versus a group receiving an anabolic steroid (nandrolone) in terms of physical activity outcomes in terms of the AAS^36^. It is possible that combined exercise for a longer time (16 weeks) had a higher impact on daily physical activity.

In terms of physical function, a significant group–time interaction was found only for the OLST, while the OLST time also increased in the ID group. To our knowledge, ours is the first study to use the OLST to measure physical functioning in a population on HD. The fact that balance improved only in the ID group is surprising because the ID program did not include any exercise that specifically targeted this ability. However, this outcome may be explained by the improved lower limb muscle activity generally associated with exercise and the higher exercise adherence rate in the ID program^37^. This result, since is the only functional measure that showed a group per time interaction effect, could also be found by chance.

The impact of exercise programs on the other physical function metrics was not significantly different between groups. Thus, like the physical activity level outcome, the functional level of the participants improved in both programs. The STS-10 results improved from baseline to 16 weeks although we did not achieve the minimal detectable change (MDC) of 8.4 seconds^38^. The results for the ID group concur with those from previous studies reporting improvements in this test ranging from 2.5 to 5.75 seconds^8,9,18,39–41^. However, the magnitude of improvement in the HB group was less than in a previous study^18^. Compared with these previous studies, our patients required more time at baseline to perform the tests. Moreover, poor adherence in the HB group may have negatively affected our results.

The 6MWT results significantly improved between the baseline and 16 weeks, even though the MDC of 66.3 meters was not achieved^38^. We detected a similar change in the ID group as in previous studies^42–44^. Nevertheless, the improvement in the HB group we achieved was lower than in previous studies^19,20^, perhaps because these participants had used a pedometer for walking training, thus providing them with direct feedback which could have been a motivational factor. The 6MWT increased in one study^28^ that implemented walking training in a home-based program. This study ensured the education of the dialysis personnel in the exercise program, who also assisted in the training phase of the trial. Thus, the nursing team could encourage patients to complete the program and this fact could have resulted in increased adherence to exercise.

In agreement with a previous study^18^, after 16 weeks of ID or HB training neither the group–time interaction or time significantly affected self-reported HRQoL (KDQoL-36). There was a non-significant physical component improvement over time. Other studies that evaluated the HRQoL via the SF-36 also revealed inconsistent results, perhaps because of the different exercise program durations, the variety and intensity of exercise types used, and the different characteristics of the participants in each study.

Although we did not find significant mental composite results in this study using the KDQoL, the CES-D–time factor did significantly change (*p* = 0.017). Previous studies measuring depression with others questionnaires (such as the *Hospital Anxiety and Depression scale*^21,45^ or the *Beck Depression Inventory*^26,45^) also found a significant improvement in this variable after exercise.

Participants and clinical researches described a range of barriers to implement the exercise program to explain the low rate response and high dropout rate. Primary barriers for patients were lack of prior knowledge about safety and benefits in exercise programs, fear of injuries during exercise fatigue or symptoms of weakness, and lack of interest or motivation, all of which have been described in previous studies^15,46^. These studies also describe that if the professional responsible for the exercise program is not routinely treating the HD patients could also be a barrier to participate in exercise programs, so as the lack of explicit nurse support to encourage the exercise program implementation.

In our study, the HB participants reported that they completed only 53% of the proposed sessions and it was very difficult to convince them to exercise on their own. We found that patients did not undertake the exercise as suggested and did not implement the program in their life routine. We implemented several strategies to try to improve adherence, but none were very successful. Our HB exercise adherence rate was similar to that reported in a previous study in which the HB group completed 58% of the sessions (using a pedometer)^24^, while adherence in a study by Koh *et al*. was 71% ± 13% (using a diary)^23^. This fact underscores the essential importance of HD unit nurses and nephrologists as a means to identify and overcome barriers to exercise. In our study, the fact that the physical therapist was external to the unit was a previously described barrier^46^. Even though adherence was low, we observed an improvement in the physical condition of all the patients when they participated in a HB exercise program.

## Study Limitations

Although the findings of this study are positive for both the ID and HB groups, some limitations should be acknowledged. We had a high number of dropouts in both groups and some participants did not want to exercise in the group to which they were randomly allocated: the majority preferred the ID exercise training format. Most of the participants from the HB group did not have support at home and were not supervised or supported by a relative. The only motivational support that they had was when the physical therapist approached them. Moreover, the difficulty level of the strength exercises in the ID group may have been insufficient, even though we measured the 10 repetition maximum every two weeks to ensure their resistance training progression.

## Methods

### Study design

This was a randomized controlled clinical trial. Eligible participants were randomly assigned either to the ID or HB exercise group. The exercise program lasted 16 weeks and the patient metrics were recorded at baseline and after the intervention.

### Participants

The study participants were recruited from two HD units in Valencia (Spain) and were assessed for eligibility by the consulting nephrologist at each unit. For inclusion the patients had to have a stable medical condition and have been receiving HD therapy for at least 3 months. The exclusion criteria were as follows: (a) myocardial infarction in the 6 weeks prior; (b) cardiovascular disease that could worsen with exercise; (c) above the knee amputation without a prothesis; (d) cerebral vascular disease; (e) musculoskeletal and respiratory disorders that might worsen with exercise; and (f) inability to complete functional testing.

This research was approved by the Ethics Committee at the *Hospital Universitario Doctor Peset* in Valencia (Spain; registration number 1/15). Participants provided their written informed consent to participation. This study respected the fundamental principle set out in the Declaration of Helsinki. It met the requirements set out in legislation in accordance with the Data Protection Act. This study was registered at Clinical Trials (NCT02832440; date of registration 08/07/2016).

Participants were randomized by an external investigator in blocks, 4 codes per block, based on age and sex simultaneously, either into the ID or HB group using the www.randomization.com webpage. The allocation was concealed and the researchers performing the assessments were blinded to the random assignment throughout the whole study period.

### Intervention and adherence

**Figure 2:**
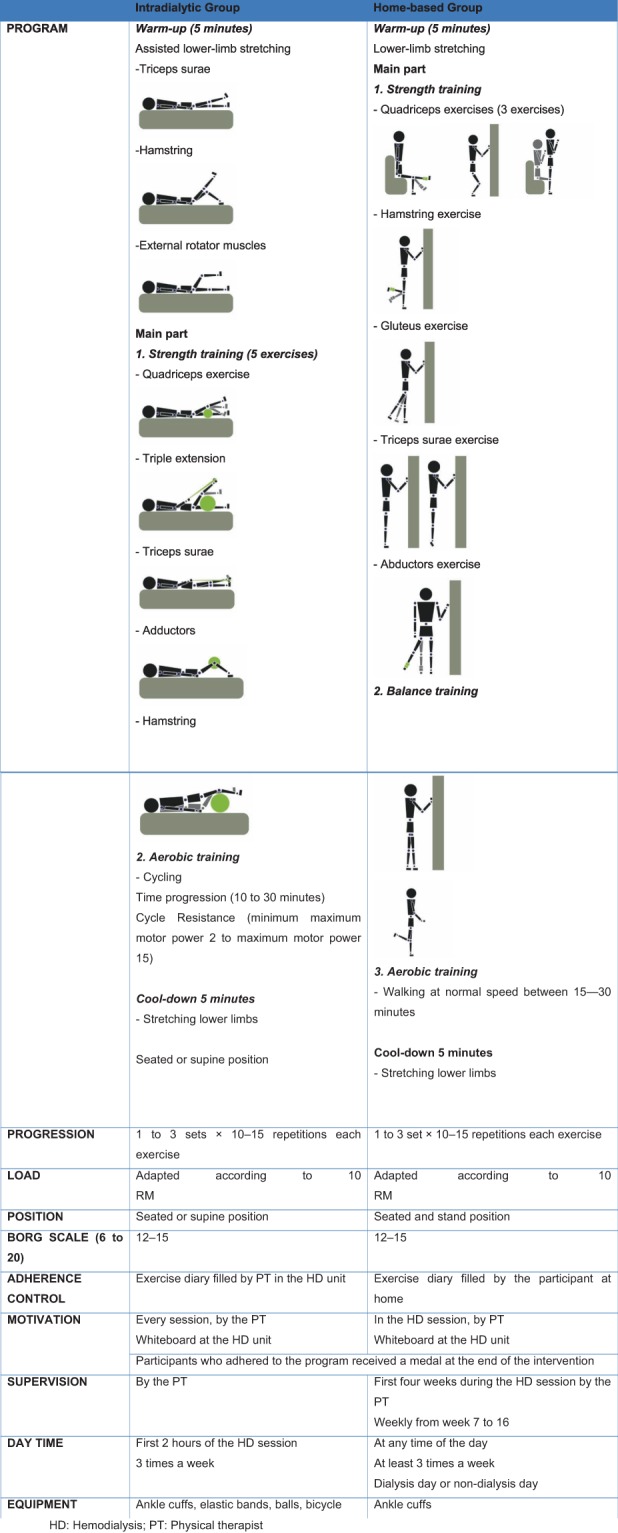
The exercise intervention completed by both the intradialytic and home-based trial groups. HD: Hemodialysis; PT: Physical therapist.

Both the ID and the HB exercise interventions lasted 16 weeks and are detailed in Fig. 2. Progression was achieved by increasing sets, from 1 to 3, 10 repetitions per set, and load was adapted every 2 weeks after the 10RM test, so that it progressed from 1 kg to 17 kg in some cases. We used ankle cuffs of different weights (1 to 4.5 kg) and elastic bands of increased resistance. In the ID we used the cycle ergometer Mottomed Letto to work the aerobic part (10 to 30 minutes), while the HB were instructed to walk in their normal speed from 15 to 30 minutes. Intensity was adjusted by the Borg Rating of Perceived Exertion Scale at level between 12 and 15. The programs were elaborated by a physiotherapist specializing in therapeutic exercise and aimed to work similar lower-limb muscle groups. Treatment adherence was defined as the number of sessions performed divided by the number of sessions offered, multiplied by 100.

### Outcome measurements and tools

Patient clinical and anthropometric data were collected. Physical function was assessed with a battery of functional tests at baseline and after 16 weeks. Physical therapists who were blinded to the individuals’ allocation assessed the participants immediately before their HD session. To standardize the procedure, we followed a previous study that developed a detailed script for all the functional tests^47^.

As primary outcome we evaluated patient physical activity levels using the *Human Activity Profile* (HAP) questionnaire which has been validated in populations with CKD^3^. The HAP comprises a list of 94 activities each assigned a number relating to the vigorousness of the task and the participants must report whether they still do this activity, no longer perform the activity, or never did the activity. The HAP assesses the maximal activity score (MAS; the number assigned to the activity with the highest oxygen consumption requirement that the patient still performs) and the adjusted activity score (ASS; the difference between the MAS and the number of activities with lower scores—i.e., less demanding tasks—that the participant has stopped performing). Depending on the AAS, individuals can be classified as having impaired activity (AAS < 53), being moderately active (AAS = 53–74), or as active (AAS > 74)^48^. This questionnaire, especially the AAS, has a high test-retest reliability in this population (MAS intraclass correlation coefficient [ICC] = 0.76; ICC ASS = 0.92)^49^.

### Secondary outcomes

Prior to the first weekly HD session, participants performed the *Short Physical Performance Battery* (SPPB), one-leg standing test (OLST), and timed up-and-go (TUG) test. The SPPB is a battery of functional tests that assesses balance in three different positions (side-by-side, semi-tandem and tandem) for 10 seconds each; gait speed in 4 meters at normal pace; and ability to stand up from a chair 5 repetitions. The score ranges from 0 to 12. The OLST measured the time that each participant could keep a one-leg balance position with eyes open and allowing free movement of the arms. The participants were given 3 trials to try to achieve 45 seconds. The TUG measures the best time of two consecutive trials in standing up from a chair, walking 3 meters, turn back and sit down again. During the second weekly HD session, the sit-to-stand 10/60 (STS-10/60) one-leg heel raise (OLHR), and handgrip strength (HG). The STS-10 measures the time required to complete 10 consecutive full stands from a sitting position, and the STS-60 measured the number of repetitions of sitting down on and getting up from a chair achieved in 60 seconds. The OLHR measures the functional strength of the triceps surae muscle in each leg. The handgrip strength was used to measure the amount of strength developed by each hand, three consecutive repetitions were recorded and the highest peak force was recorded. Finally, in the third weekly HD session the 6-minute walking test (6MWT) was performed and assess the longest distance that a patient can walk during 6 minutes, using any ambulation aid if required. Further detail of the measuring protocol, so as the reliability of all these tests has been reported in previous studies (ICC = 0.83–0.97)^38,50^.

To evaluate the physical activity level, the participants also completed the *Physical Activity Scale for Elderly* (PASE) questionnaire which has been validated a population with CKD^3^. The PASE assesses the time spent in the week prior doing specific activities commonly performed by elderly individuals. The total score is recorded as the sum of the amount of time that the person spent on each activity (hours per week) multiplied by the weight assigned to each activity^51^.

To assess the symptoms of depression, the participants completed the *Center for Epidemiologic Studies Depression* (CES-D) scale, which comprises 20 questions about depressed mood. Individuals respond to the CES-D by rating the frequency with which they experienced each mood in the week prior on scale ranging from 0 to 3. The total score ranges from 0 to 60 points with higher scores indicating the presence of more symptoms of depression. Participants undergoing HD with a score of 18 or more points were considered at risk for depression^52^.

To assess HRQoL in patients with CKD, we used the *Kidney Disease Quality of Life-Short Form* (KDQoL-36) survey which comprises 36 items that describe the individual’s perception of their health in the 4 weeks prior. It is divided into five categories: (i) symptoms/problems; (ii) kidney disease burden; (iii) kidney disease effects; (iv) physical components; and (v) mental components. The maximum score per category is 100 points and higher scores indicate better perceived health status^53^. This Spanish version of the questionnaire has been validated and is reliable, with ICCs ranging from 0.62 to 0.77 for each dimension^4^.

### Sample size calculation

The sample size was calculated by detecting changes in physical activity using the average AAS HAP score. Taking previous data from an exercise-based intervention in HD patients into account^54^, and considering an alpha error of 0.05 and a statistical power of 80%, a total sample size of 26 participants were required to detect an effect size of 0.288 (G*Power, ANOVA: repeated measures, within–between interaction). We should add 20–30%% for dropouts that happen in most of the studies in this cohort, and then we should have included 31 participants at least. Since the effect size used (from previous data) is low, we consider this study to be a pilot trial.

We performed a post-hoc power calculation. Considering an effect size of 0.17, alfa 0.05, total sample size 23, 2 groups and 2 measurements, the power achieved was 0.34 (G*Power, ANOVA: repeated measures, within–between interaction).

### Statistics

The normal distribution as well as the skewness and kurtosis of the data was checked using the Kolmogorov–Smirnov test. Results are presented as the median or mean (± the standard deviation [*SD*]). The Mann–Whitney U test was used to check differences between groups since the data did not follow a parametric distribution at baseline. Differences on categorical variables were checked with the Chi-Squared test. To analyze the effect of the exercise programs we used the ANOVA mixed model with time as the within-group factor and the exercise group as the between-group factor. A per-protocol analysis was performed instead of an intention to treat analysis. The SPSS package, version 20.0 for Windows (IBM Corp., Armonk, NY) was used for all the statistical analyses, results were recognized as statistically significant at a threshold cut-off of *p* ≤ 0.05 for all the statistical analyses.

## Conclusion

There was no difference between a combined 16-week aerobic and muscle strength exercise program and a home-based program on physical activity levels and physical functioning (besides OLST). Both interventions were associated with positive changes of the outcomes
